# Making the invisible visible: results of a community-led health survey following PFAS contamination of drinking water in Merrimack, New Hampshire

**DOI:** 10.1186/s12940-019-0513-3

**Published:** 2019-08-30

**Authors:** Bindu Panikkar, Benjamin Lemmond, Laurene Allen, Carol DiPirro, Shaina Kasper

**Affiliations:** 1 Rubenstein School of the Environment and Natural Resources, 81 Carrigan Dr., University of Vermont, Burlington, VT 05405 USA; 2 Merrimack Citizens for Clean Water, 16 French Court, Merrimack, NH 03054 USA; 3 Toxics Action Center, 141 Main St., Suite 6, Montpelier, VT 05602 USA

**Keywords:** Per- and polyfluoroalkyl substances, Perfluorooctanoic acid, Perfluorooctane sufonate, PFAS, PFCs, Drinking water contamination, Environmental health, Environmental justice, Community health survey

## Abstract

**Background:**

In March 2016, citizens of Merrimack, New Hampshire, learned that their public water supply was contaminated with perfluorooctanoic acid (PFOA). A subsequent state-led investigation revealed widespread contamination of both public and private well water with PFOA and several related chemicals, broadly termed per- and polyfluoroalkyl substances (PFAS). This research examines the local response to PFAS contamination of the public water system and well water in Merrimack and the results from the health survey administered by a local advocacy group, Merrimack Citizens for Clean Water (MCFCW).

**Methods:**

MCFCW designed and implemented a community health survey (*n* = 596) representing 213 households exposed to PFAS through drinking water. The surveys were conducted in the summer of 2017. Respondents used an online survey platform to report demographic information, exposure sources, and health conditions. Logistic regression was used to analyze the community-based health survey results .

**Results:**

There were several important associations that warrant further investigation and more immediate attention, especially: 1) elevated incidence of developmental, autoimmune and kidney disorders among those under 18 years of age; 2) elevated levels of health concerns, multiple health concerns, autoimmune disorders, and reproductive disorders among women, 3) elevated levels of health concerns, multiple health conditions, cardiovascular, respiratory, reproductive, and liver disorders in those with industrial occupational exposures, and; 4) elevated incidence of health concerns, cardiovascular, and developmental disorders among those who have been living in Merrimack for a long time versus newer residents.

**Conclusions:**

The limitations inherent in the study design warrant caution in interpreting the results, however the associations found in this study merit further investigation. This health survey highlights foremost the critical gap in information—lack of access to blood testing, medical monitoring and physician guidance of PFAS-exposed residents. This study provides a model for conducting community-based health studies to advocate for pathways to state supported biomonitoring and medical monitoring for those exposed to industrial toxins and to take into consideration the human health burden in shaping the future of chemical regulation.

## Introduction

In February 2016, managers at the Saint-Gobain Performance Plastics plant in Merrimack, New Hampshire, reported to state officials that a toxic chemical called perfluorooctanoic acid (PFOA) had been detected in the tap water at the plant facility at a level of 30 parts per trillion (ppt) [1]. This finding was concerning, since the water that the plant used was sourced from the Merrimack Village District (MVD) public water supply. A subsequent New Hampshire Department of Environmental Services (NHDES) investigation found levels of PFOA in MVD wells that supplied the towns of Merrimack and nearby Bedford, NH, at levels ranging from 17 to 90 ppt [2]. Two contaminated MVD wells were deactivated and additional testing of municipal and private wells was conducted throughout Merrimack and Bedford. As of June 29, 2016, 527 wells had been tested for PFOA and a related chemical, perfluorooctane sulfonate (PFOS) [1]. About 30% (166 wells) showed these chemicals in excess of the recently established Environmental Protection Agency (EPA) Lifetime Health Advisory (HA) level of 70 ppt for both chemicals individually or combined [3–5]. The residents of Merrimack and Bedford were faced with trying to understand the implications of what one NHDES official described as “by far the largest groundwater investigation ever undertaken in the state” [6], and the complex and uncertain potential health consequences of exposure to these chemicals. This research identifies gaps in knowledge and “undone science” left by the state’s response to PFAS contamination in Merrimack and analyzes the results of a citizen-led health survey completed by nearly 600 exposed residents.

## Background

In 1984, a company called ChemFab purchased a former General Electric plant in Merrimack and began manufacturing its brand of chemically weatherproofed fabrics, which were often used in large installations such as military tents and stadium roofing [7]. ChemFab operated similar manufacturing facilities in Bennington, VT, and Hoosick Falls, NY. In 2000, ChemFab was purchased by the French manufacturing conglomerate Saint-Gobain, and in 2002, Saint-Gobain shuttered its facility in Bennington, VT, and boosted its manufacturing operations at the Merrimack plant under the name Saint Gobain Performance Plastics (SGPP).

The primary chemical component of the mixtures ChemFab/SGPP used to coat their fabric products is polytetrafluroethylene (PTFE), best known by its trade name, Teflon®. PTFE is a synthetic chemical with exceptional resistance to heat and chemical solvents. The PTFE-based dispersions that ChemFab and SGPP used to coat fabrics, at least until 2015, also contained a chemical called ammonium pentadecafluorooctanoate (APFO) [8], an ammonium salt of PFOA that dissociates into PFOA in the environment [3]. APFO/ PFOA belongs to a large class of synthetic chemicals known as per- and polyfluoroalkyl substances (PFAS), which share the chemical structure of a chain of fluorinated carbon atoms with various functional group attachments. These chemicals first generated headlines in the early 2000s when they were found to have contaminated the drinking water for 70,000 people of Parkersburg, WV, where a DuPont plant made Teflon and related products for decades [9]. The EPA investigation into Dupont and subsequent legal settlement resulted in the largest epidemiological study of PFOA toxicity to date, known as the C8 study (C8 is another name for PFOA, which has a chain of eight carbon atoms). The C8 study investigated links between PFOA and dozens of health effects, and determined “probable links” between PFOA exposure and health effects (detailed below). Since the C8 study, the EPA worked with manufacturers to voluntarily reduce and eventually eliminate PFOA and PFOS in their supply streams [10]. Even as PFOA and PFOS are being phased out due to toxicity concerns, in many cases they are being replaced by other PFAS with slightly different chemical structures [11]. The number of sites where PFOA and other PFAS chemicals are now found in soil and groundwater is still growing, particularly in former military, municipal, and industrial sites where they were produced or used [12].

At ChemFab/SGPP facilities in New England, dispersions containing PFAS were used for decades under the presumption of safety, as recounted by former ChemFab workers who handled dispersions and other raw materials with little or no personal protective equipment [13]. In the 1980s, concerns began to emerge over the air emissions at ChemFab’s Bennington facility as residents began complaining about odors emitted from the plant. The company pushed back against attempts by Vermont regulators to require devices called abaters, which incinerate chemicals present in stack emissions, in ChemFab’s stacks. One strategy the company used was to repeatedly threaten to move operations to its Merrimack facility if Vermont regulators continued pressing for abaters [14]. Soon after the takeover of ChemFab by Saint-Gobain, the company closed its Bennington plant in 2002 and in turn increased its operations in Merrimack. Though it is not certain that environmental regulations were the deciding factor in this move, and while ChemFab faced virtually no punishment for its repeated violations of existing environmental regulations in Vermont [15], one Bennington plant manager reported then to a local paper that looser pollution controls in New Hampshire was one of the reasons the company moved its operations there [14]. Even as the Bennington site and another ChemFab site in Hoosick Falls, NY, have been implicated in PFAS groundwater contamination in recent years, New Hampshire remains the least protective of the three states in terms of health advisory and drinking water standards for PFOA and PFOS. This difference adds an environmental justice dimension to the New Hampshire contamination, as it creates the possibility that New Hampshire residents were exposed to higher PFAS pollution levels for longer as a result of differences in regulatory standards—both prior to and in the aftermath of the discovery of PFAS contamination in the Merrimack area.

Exactly how much PFAS were emitted by ChemFab/SGPP over the decades of its operations is not known. However, it is clear that the dispersions used at the facility at least as early as 2004 contained APFO, based on the company’s disclosures to regulators [8]. In 2005 the facility was cited for violating air emissions standards for APFO, and as part of a consent decree with NHDES, SGPP committed to using dispersions with reduced APFO content in 2006 [16]. SGPP has since claimed that dispersions currently used contain no APFO [1]. Testing in 2016 and 2018 nonetheless showed that several PFAS, including PFOA, were still present in stack emissions from the facility [17–19]. Other releases of APFO/PFOA occurred over the years from on-site spills and waste disposal, according to communications between SGPP and NH state officials, which note a number of spills and describe how wastewater from washing equipment and drains from sinks at the Merrimack facility went directly into the municipal sewer from 2002 until 2015, when the company began hauling wastewater off-site for disposal [8, 20]. Although as many as forty different PFAS have been detected at the SGPP site, the state’s investigation and remediation efforts have largely focused on PFOA and PFOS [21].

Notably, for most of the time that ChemFab/SGPP used PFAS-based dispersions, New Hampshire did not have groundwater or drinking water standards in place for any PFAS. New Hampshire only adopted groundwater standards for PFOA and PFOS in 2016 after the EPA released its lifetime Health Advisory levels of 70 ppt for both chemicals individually or combined [22]. Groundwater testing for PFAS in Merrimack or other ChemFab/SGPP sites was never mandated by state or federal agencies for small water systems like MVD prior to the discovery of the contamination. While SGPP has released the formulas for the seven dispersions it used at the Merrimack facility beginning in 2004, the company’s lawyer declined to share historical production rates, calling the request for such information “vague, ambiguous, and overbroad” because the “Merrimack facility’s volumetric usage of a particular chemical … does not correlate to a certain number of discharges of that chemical” [8]. While ongoing monitoring of public and private wells has provided details on PFAS levels in water since 2016, there is no data from environmental media, drinking water, or biomonitoring studies prior to 2016 to show what levels residents were exposed to over the decades of SGPP and ChemFab’s operations.

### Local and state response to PFAS contamination

The first public announcement by the NHDES of the presence of PFAS in drinking water in Merrimack came in the form of a press release, on March 4, 2016, announcing the finding of PFOA at “low levels” of 30 ppt [23]. A series of public meetings followed on March 23 and 24, after the agency had conducted some initial groundwater testing [1]. At those meetings, residents learned that PFOA had been detected in private wells at levels ranging from 17 to 820 ppt, and water in wells that supplied MVD ranged from 17 to 90 ppt PFOA. While there were no regulatory limits then on groundwater concentrations of PFOA or other PFAS, the EPA had set a provisional Health Advisory Level of 400 ppt in drinking water—which amounted to an unenforceable recommendation to state and local regulators, rather than a legally binding regulation. Meanwhile, several neighboring states were working on more protective advisory levels and in some instances setting enforceable regulations. While public meetings were taking place in Merrimack, Vermont had already adopted a Health Advisory Level for PFOA of 20 ppt [24], and the New Jersey Drinking Water Quality Institute was developing recommendations on enforceable Maximum Contaminant Level (MCL) and groundwater standards for several PFAS [25]. New Jersey regulators have since adopted an MCL for one PFAS (13 ppt for perfluorononanoic acid or PFNA), and new groundwater standards for PFOA (14 ppt) and PFOS (13 ppt) [26].

At the March 23 meeting in Merrimack, NHDES officials announced they would provide bottled water “out of an abundance of caution” to any private residence with a well where PFOA/PFOS was found at or above 100 ppt [27]. Representatives from state agencies then repeatedly assured residents that MVD water was safe. A presentation by the State Epidemiologist, Dr. Benjamin Chan, stressed the inconclusiveness of the science linking PFAS exposure to health outcomes and finished with the suggestion that residents not seek out blood testing, except for those residents living within one mile of the SGPP site with wells testing above 100 ppt who wanted blood tests.

Residents at the meeting, however, had many questions and doubts about using the water – in particular those with health conditions and those with small children [27]. Was water under 100 ppt actually safe? How long had they been exposed to these chemicals, and what were the levels before? Most residents voiced concern and frustration over the state’s reassurances of the safety of the public water when there was still so much uncertainty about the health effects of PFAS. Appeals were made by the residents for the state to work with the Agency for Toxic Substances and Disease Registry (ATSDR) and other federal agencies to study the health consequences of contamination.

The state’s response to the contamination, however, largely focused on documenting environmental contamination and negotiating with SGPP on water treatment, bottled water, and infrastructure upgrades, placing less emphasis on the question of health studies. The state’s groundwater investigation continued, and by the end of March 2016, 107 wells had been tested, with results in the range of 0-830 ppt; in total, 26 private wells exceeded the 100 ppt action level for bottled water provision [28]. On April 1, NHDES commissioner Tom Burack sent a letter to the company that stated that SGPP was “potentially responsible for the cleanup of the Site, restoration of impacted groundwater and drinking water, other actions necessary to protect public health and the environment, and any costs NHDES incurs for addressing the impacts of this contamination” [29]. In subsequent correspondence, SGPP agreed to install Point-of-Entry-Treatment-Systems to residences within 1 mile of the plant that were not on public water. Though SGPP ultimately ended up paying for water line extensions, bottled water, and treatment systems for over 750 properties in Merrimack, Litchfield, Bedford, and Manchester, the company never admitted liability for the contamination. The dispute of who is ultimately responsible for the contamination remains unsettled, and there are several legal cases over liability pending in court [30].

By May 2016, the radius of homes eligible for testing and bottled water had expanded from one mile from the SGPP site to 1.5 miles, and the cutoff concentration for receiving bottled water had been lowered from 100 ppt to 70 ppt, following the announcement of a new EPA lifetime Health Advisory level of 70 ppt for both PFOA, and PFOS. By the end of June, 527 wells had been tested, with 166 (31%) showing contamination above the new 70 ppt cutoff [1]. These repeated changes in advisory levels and cutoff limits for bottled water or testing, from 400 ppt to 100 ppt and 70 ppt, coupled with the adoption of more stringent limits by other states, left community members unsure of what was truly “safe” and further undermined trust in state agencies that the levels being set were actually protective of health.

Following the water testing, blood levels of some residents were also tested, though the initial tests were only offered to residents living within the 1.5-mile radius and with private wells testing of over 70 ppt. In September 2016, several residents presented the Merrimack Town Council with a petition to expand blood testing to public water customers, among other demands [31]. The New Hampshire Department of Health and Human Services (NHDHHS) announced that month that it would include MVD customers as part of an ongoing PFAS blood testing project that the agency had been conducting in Pease Tradeport, a former military site near Portsmouth, NH. Portsmouth has population of 21,485, only a small fraction of whom live near Pease Air Base; the blood testing at the Pease site included 1578 individuals. Merrimack is a town of over 25,000 residents—87% of whom are served by MVD water [32]—still, NHDES set a target of just 200 individuals as a representative sample. A total of 217 residents participated, from 132 households. At a public meeting in October 2017, a NHDHHS representative said that they were aware that more residents wanted blood tests, but testing up to 5000 residents would be prohibitively expensive, likely costing over a million dollars [33].

In petitions to the town council and presentations at various town and district meetings, citizens made the case for immediate filtration of drinking water in all public schools, for accelerated preparation of filtration designs for all public wells, and expanded blood testing available to all residents [31]. Comments poured in on online petitions and public forums from community members who were troubled by diagnoses of illnesses with no family history, in particular autoimmune illnesses. At a public meeting in March 2017, some town councilors pushed back against the notion that the town’s water supply was contaminated, stating that PFAS were ubiquitous on the planet and that using such language was a “scare tactic” [34]. Once again, the inconclusiveness of the science around PFAS exposure was cited as a reason not to raise alarm about PFAS in public water and health outcomes.

The results of the MVD public water user group blood tests, released in October 2017, showed that the MVD group had blood levels of PFOA over twice the background levels detected in the U.S. population in the most recent screening; they also indicated that proximity to the SGPP facility, age, and tap water consumption all contributed to higher serum PFOA levels [35]. (Blood tests of residents with private wells were not released to the public.) Blood serum levels of PFOA in the MVD group were on average 3.9 μg/L (or 3900 ppt), with a 95th percentile level of 10.1 μg/L. Average serum PFOA levels in the U.S. population (sampled in 2013–4) were 1.9 μg/L, with a 95th percentile of 5.6 μg/L. Among the MVD customers sampled, serum PFOA levels were greater for those living less than 1.5 miles from SGPP versus those living outside the 1.5 mile radius (mean serum PFOA level of 6.3 μg/L versus 5.9 μg/L), and between the older age groups compared to younger age groups (4.8 μg/L for those over 60 years of age versus 3.2 μg/L for those under 19 years of age). Serum PFOA levels were also higher for MVD customers who drank more cups of tap water per day, with an average serum PFOA of 4.7 μg/L for those who drank 8 or more cups a day versus 3.2 μg/L for those who drank 0–3 cups a day. However, no significant difference in serum PFOA was found between groups of people living at their residence for either more or less than 10 years.

While the analysis of the blood results was ongoing, NHDHHS also moved to conduct a cancer incidence report of the Merrimack area. The report, released in January 2018, compared rates of 26 kinds of cancer in Merrimack and nearby areas over a period of ten years ending in 2014 to background levels reported in the state. Despite higher than expected cancer rates, no significant elevation  was determined in Merrimack’s cancer rates [36]. NHDHHS has not conducted other assessments of non-cancer health effects related to PFAS exposure, even though non-cancer effects such as blood cholesterol level and lowered immune response are more strongly and consistently linked to PFAS exposure [37–40].

Throughout this process, many Merrimack residents became increasingly frustrated with the state and local agencies’ handling of the crisis, and felt that these agencies were dismissive of their concerns. In particular, residents felt that the state’s response had failed in three critical ways: 1) to act to protect sensitive populations through filtration for schools and through more protective action levels (i.e., cutoff levels for bottled water provision) and regulatory standards; 2) to expand blood testing to a wider group of the exposed population; and 3) to track a wider spectrum of health effects related to PFAS exposure, beyond cancer incidence. As discussed below, there are many indications of sensitive endpoints associated with PFAS exposure including but not limited to cancer.

### Health effects of PFAS

PFOA and PFOS are part of a large group of synthetic chemicals called per- and poly-fluoroalkyl substances (PFAS). PFAS are ubiquitous in food [41, 42], water [43], and biological samples of humans worldwide [44, 45]. Ingestion of food and water and inhalation of PFAS-containing particulates (e.g. soil, dust) are the most significant exposure pathways for humans [38]. Some people are exposed to higher levels of PFAS through occupational exposure and proximity to contaminated areas [46], such as people working in factories that produce/use these compounds, or communities living close to facilities using PFAS in large quantities (e.g. military bases) [43].

PFOA and PFOS are extremely durable and persistent chemicals that do not break down easily in the environment. While PFOA and PFOS are not particularly volatile, their transport and detection in remote places may result from the transport of volatile perfluoroalkyl acid precursors [47, 48]. PFOA and PFOS are not easily removed by municipal wastewater treatment [49, 50], and may even increase in concentration as polyfluorinated perfluoroalkyl acid precursors are converted to perfluoroalkyl acids in the treatment process [51, 52]. Further environmental releases can occur from applying treated effluents and biosolids back to the land [53]. Some PFAS have been shown to bioaccumulate in blood, though the biological fate of all PFAS has not been well studied [54].

Numerous health endpoints have been associated with PFOA and PFOS exposure. Occupational health studies conducted by the chemical industry on workers documented associations between PFOA levels and higher rates of mortality from prostate and bladder cancer as well as changes in cholesterol and increased levels of estradiol, a sex hormone [37]. The C8 study of health risks to the communities near a DuPont factory found a “probable link” between PFOA exposure and kidney cancer, testicular cancer, ulcerative colitis, thyroid disease, pregnancy-induced hypertension (including preeclampsia), and hypercholesterolemia [55–57]. Elevated levels of serum PFOA and PFOS at exposure levels seen in adults throughout the US have been associated with increased cholesterol [58], reduced fertility [59], and thyroid disease [60]. PFOA in mothers’ blood have been linked to slightly lower birth weights and/or head circumference in their babies [56–58], weakened immune system in children [61], and birth defects [62]. Some studies show elevated levels of certain PFAS in the blood of children compared to adults [63, 64]. A recent review of health effects of PFAS exposure in children found consistent associations between PFAS levels and several children’s health endpoints including age of menarche in girls, vaccine response, and renal function [65]. Additionally, animal studies have linked PFAS with harm to multiple organ systems in multiple species, with changes to the liver, spleen and kidneys, weakened immune system, delays in maturation and an increased risk of death during weaning compared to unexposed controls [37]. PFOA has also been linked to cancer in rats and altered mammary gland development in mice [37].

There remains some uncertainty over the precise mechanisms by which PFAS affect human health, and the exact dose-response relationship for various PFAS [66]. Also, while many chemicals are studied individually, the effects of exposures to a mixture of PFAS is largely unknown [65–68]. While there are consistent findings for effects of exposure to PFOA in particular and endpoints such as increases in cholesterol, changes in certain liver enzymes, increased uric acid in blood serum, decreased fetal growth, and decreased vaccine response [40], conflicting or inconclusive results from studies on other health endpoints makes it difficult to draw firm conclusions about the full range of health impacts of PFAS exposure. More research is needed to define the precise dangers of PFAS exposure to better protect against harmful effects from these chemicals. In addition to scientific uncertainty, political divisions have impeded efforts to communicate the health risks of PFAS. Reports note that the EPA delayed a report from the ATSDR that had calculated safety levels several times lower than the EPA’s 70 ppt advisory level, based on more sensitive endpoints such as immunotoxicity [69, 70]. The ATSDR report also showed toxicity concerns for two additional perfluorinated compounds, perfluorononanoic acid (PFNA) and perfluorohexane sulfonic acid (PFHxS).

Science and research is integral to social movements and environmental health and social justice issues. High-quality scientific information is increasingly important in matters of remediation and compensation in a legal setting. Conducting high-quality studies of health consequences of environmental exposures, however, is expensive and time-consuming. This creates a situation of “undone science”, and perpetual lack of information on health impacts from environmental exposures [71]. There exists an urgent need for community based health studies to be conducted in a timely manner to reduce risks. In the systematic absence of science or undone science, or underfunding of topics identified by the public, the community themselves are tasked with proving the health associations that they suspect from harmful exposures. The community-led health survey examined in this research is a response to undone science to define the risks and hazards faced by the residents in Merrimack. The survey represents a community-led alternative to reliance on state and federal agencies to define and study environmental problems.

## Methods

Starting in May of 2016, a group of Merrimack residents began meeting regularly to discuss the water contamination situation and possible avenues of response. By early 2017, residents officially formed Merrimack Citizens for Clean Water (MCFCW) (https://www.cleanwaternh.org/), a group that advocates for remediation of water contaminants, health monitoring of exposed populations, stricter regulatory standards on PFAS, and increased transparency from state and local regulators. MCFCW made repeated attempts to engage state agencies to expand blood testing and to study the health effects of PFOA exposure in the community, however, their requests for further agency involvement in health tracking never materialized. Members of the group felt that the official health study was not coming anytime soon, and if they did not do anything, reports of health effects that were shared in local forums would never be documented or studied (personal communication).

In March 2017, MCFCW presented the Merrimack contamination case at a Boston University School of Public Health workshop, which was co-sponsored by the Toxics Action Center (TAC), a New England-based nonprofit that works with communities affected by toxic contamination. Soon after, MCFCW began designing a health survey for Merrimack residents called the “Merrimack PFOA Concerns Health Survey” to document and study potential impacts of PFOA exposure in the Merrimack area. They felt that conducting a health survey gave voice to those who felt invisible and abandoned by the state response [72]. Greg Howard at the Boston University School of Public Health provided feedback on survey design and methods; TAC provided technical support throughout the process. The survey gathered three kinds of information: (1) basic demographic profiles of respondents (age, gender, years living in Merrimack, occupational history with ChemFab/SGPP, etc); (2) information on exposure variables such as water source, water filtration, and participation in the DHHS blood test program (indicative of proximity to the SGPP site); and (3) self-reported occurrence of certain health conditions. Links to additional information about PFAS and health were provided at the end of the survey.

The survey was created on a Google Forms platform, which participants could access by a URL. Most respondents completed the survey online, with a few conducted by phone. Participation was sought by posting flyers in the community, press releases in local papers (The Nashua Telegraph, the New Hampshire Union Leader, and the Merrimack Journal), postings on relevant social media sites (e.g. the Merrimack Water Issues Facebook Forum, the Merrimack Town Facebook Forum and the Positive Merrimack Facebook Forum). Surveys were also distributed door-to-door by volunteers in parts of Merrimack. The survey informed participants that no personally identifiable information would be made available to the public or to media. The participants were also informed that the results would be shared as a group dataset with the public and NHDHHS. No institutional IRB approval was sought for the study, as it was conducted by community members without any involvement with academic partners.

MCFCW representatives presented the preliminary survey results to community members and representatives from NHDHHS in August and September 2017, respectively, and also reached out to ATSDR representatives. Results were presented as bar graphs (frequency counts of health conditions). MCFCW representatives, in presenting the results, noted that they were looking for an academic partner to help with the analysis. Community members were both alarmed by the number of health conditions reported but also encouraged by the efforts to document their health concerns (personal communication). While NHDHHS representatives agreed to meet with MCFCW to discuss the health survey, they did not offer assistance with data analysis or initiate an official health study or further biomonitoring as a result (personal communication).

In total, 213 households and 596 individuals were represented in the survey over three months in the summer of 2017. The survey response rate represents over 2 % of Merrimack’s population. The survey was done by adults over 18, as confirmed through the age of the initial household respondents for all surveys. The initial respondent filled the survey for each of the household members living there including children in the house.

**Fig. 1:**
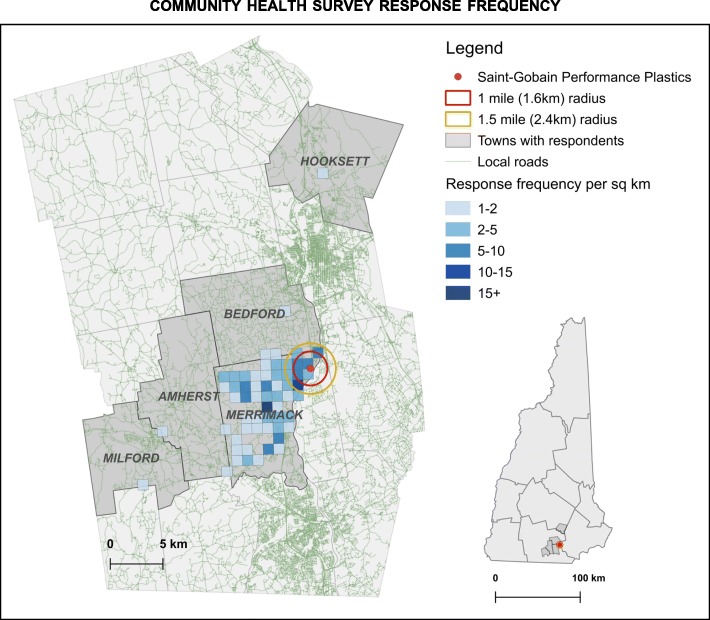
Community health survey response frequency by km^2^

The completed surveys were analyzed in partnership with environmental health researchers from The University of Vermont. Community members shared a de-identified and anonymous dataset with academic researchers for the analysis. Address-only data were used to create a coarse-scale (1 km^2^) response frequency map (Fig. 1), which shows the general geographic pattern of survey response while obscuring the exact locations of survey respondents. The community members informed many components of the analysis, including categorization of the health outcome variables. Since participants were asked to report health issues from a checklist as well as in an open-ended ‘other’ option, the raw data contained a large number of health effects that needed to be aggregated and grouped for analysis. The groups of health outcomes analyzed include: new health concerns since living in the community, autoimmune disorders, cardiovascular disorders, respiratory disorders, reproductive disorders, developmental disorders, liver disorders, kidney disorders, mental health, allergies, cancer, Lyme disease, and cases of multiple health conditions. In order to prevent double counting, no conditions were included in two different health groups, other than the “multiple health conditions” and “cancer” group, since cancer could be of either of liver, kidney, breast, prostate, etc. For instance, ovarian cancer would be included in the “cancer” group as well as the “reproductive disorder” grouping as there are significant co-morbidities in these situations.

Statistical analyses were performed using SPSS Statistics (IBM, version 24). Initial analyses conducted include univariate descriptive analysis. Descriptive analyses included the frequencies of responses for each demographic group, exposure variable, and health outcomes. Further analysis was conducted through binary logistic regression to determine the odds ratios (ORs) and 95% confidence intervals (CIs) on all the dependent health variables. To avoid placing too much emphasis on statistical significance, we emphasize the strength of associations in our results as well [73].

## Results

### Demographics

Most of the participants in the survey were from Merrimack (*n* = 579, 97%), were women (48%) and between the ages of 19 and 64 (53%). This survey also represents a smaller population (15%) of children and youth under 18 years of age (Table 1**)**. Under 3% of all respondents, or fifteen participants, lived in neighboring communities of Bedford, Brookline, Hooksett, and Milford (Fig. 1).

**Table 1:** Characteristics of Merrimack Community Survey Participants

Merrimack Community Health Survey	Frequency (*n* = 596)	Percent
City of Residence		
Merrimack	579	97.1
Other	15	2.5
Missing	2	0.3
Gender		
Female	285	47.8
Male	241	40.4
Missing	70	11.7
Age		
< 18	91	15.3
19–44	163	27.3
45–64 y	155	26
Over 65 y	69	11.6
Missing	118	19.8
Worked at Saint-Gobain /Chemfab		
Yes	10	1.7
No	564	94.6
Missing	22	3.7
Water source		
Private Well	56	9.4
Town Water	535	89.8
Missing	5	0.8
Water filtration prior to PFAS concerns		
Yes	78	13.1
No	502	84.2
Missing	16	2.7
Years of residence in Merrimack		
1–2 y	70	11.7
3–17 y	275	46.1
18–30 y	153	25.7
Over 30 y	64	10.7
Missing	34	5.7
Participated in Department of Health and Human Services blood test program		
Yes	34	5.7
No	543	91.1
Missing	19	3.2
New health concerns since living in the community		
Yes	165	27.7
No	338	56.7
Missing	93	15.6

**Table 2:** Self-reported health concerns and conditions among survey participants

Reports of Health Concerns by Category		Frequency (*n* = 596)	Percent
Multiple health Concerns	Yes	118	19.8
	No	223	37.4
Autoimmune disorders	Yes	165	27.7
	No	176	29.5
Cardiovascular Disorders	Yes	133	22.3
	No	208	34.9
Respiratory Disorders	Yes	19	3.2
	No	322	54
Reproductive Disorders	Yes	74	12.4
	No	267	44.8
Developmental Disorders	Yes	45	7.6
	No	296	49.7
Kidney Disorders	Yes	28	4.7
	No	313	52.5
Liver Disorders	Yes	30	5
	No	311	52.2
Cancer	Yes	49	8.2
	No	292	49
Allergies	Yes	20	3.4
	No	321	53.9

**Table 3:** Strength of associations between self-reported health concerns and demographic and exposure variables

		Gender	Age (ref < 18 yrs)			Occ. Exposure	Water Source	Water Filtration	Years of residence in Merrimack (ref 1, 2 yrs)		
			19–44	45–64	> 65				3–17 yrs	18-30 yrs	> 30
New health concerns since living in the community	OR	2.09	1.346	1.354	1.266	2.353	1.128	0.837	2.609	2.588	4.274
	95% CI	1.3–3.3	0.4–4.4	0.2–8.9	0.1–15.9	0.4–12.2	0.5–2.7	0.4–1.6	1.0–6.9	0.9–7.2	1.3–13.9
Multiple Health Conditions	OR	1.724	6.495	17.656	46.559	10.002	0.549	0.929	1.366	1.218	1.404
	95% CI	1.0–3.1	1.2–34.2	1.7–188.4	1.9–1110.5	0.7–138.2	0.2–1.6	0.4–2.2	0.4–4.3	0.4–4.0	0.4–5.4
Autoimmune Disorders	OR	2.684	1.214	0.601	0.672	1.139	1.483	1.977	0.458	0.548	0.321
	95% CI	1.6–4.6	0.3–4.8	0.1–5.0	0.1–12.3	0.1–10.7	0.5–4.2	0.9–4.5	0.2–1.3	0.2–1.6	0.1–1.1
Cardiovascular Disorders	OR	0.65	0.698	0.842	0.238	2.339	2.028	0.706	3.001	3.143	6.163
	95% CI	0.4–1.2	0.1–5.3	0.1–12.6	0.0–8.3	0.2–26.3	0.6–6.4	0.2–1.7	0.7–12.5	0.7–13.4	1.2–30.4
Respiratory Disorders	OR	0.397	0.055	0.45	0.003	4.845	1.857	0.777	6148	1072	5381
	95% CI	0.1–1.3	0.0–1.5	0.0–6.1	0.0–2.1	0.3–70.1	0.2–18.8	0.1–4.0	0	0	0
Reproductive Disorders	OR	2.383	1.932	1.978	5.342	9.053	0.857	0.555	1.15	0.264	2.028
	95% CI	1.2–4.8	0.4–9.9	0.2–24.3	0.2–168.5	0.9–89.4	0.2–3.0	0.2–1.5	0.4–3.7	0.1–1.1	0.5–8.3
Developmental Disorders	OR	0.627	0.065	0	0.002	0	1.758	2.96	1.176	4.966	5.456
	95% CI	0.2–1.8	0.0–0.9	0	0.0–0.8	0	0.2–19.8	0.7–12.8	0.2–8.5	0.6–42.9	0.3–90.6
Kidney Disorders	OR	1.914	0.838	4.606	4.204	0	1.639	0.264	0.791	1.032	1.012
	95% CI	0.7–5.2	0.1–11.8	0.1–224.5	0.1–811.8	0	0.2–13.6	0.1–2.1	0.1–4.4	0.2–6.0	0.1–7.9
Liver Disorders	OR	1.159	2.189	3.21	1.923	54.967	3.135	1.059	0.892	0.135	0.498
	95% CI	0.5–3.0	0.2–27.7	0.1–117.2	0.0–299.7	4.3–692.6	0.3–33.7	0.3–4.2	0.2–3.7	0.0–0.9	0.1–3.4
Cancer	OR	0.718	4.032	62.06	639.4	4.189	0.462	0.681	1.504	0.3	0.512
	95% CI	0.3–1.6	0.2–75.6	1.8–2134.5	5.5–74,316.3	0.2–99.2	0.1–1.8	0.2–2.4	0.3–7.9	0.0–2.0	0.1–3.7

Ten participants in the survey had worked at either Chemfab or SGPP at some point in their lives, but none were current employees. Only three had worked for more than two years at the facility: one individual worked at ChemFab/SGPP for 15 years, another individual worked there for five years and another worked for two years. Three others worked at the facility for less than four months and three provided no information on when they worked at the site. Results for the occupational group are reported here, with the caveat that the sample size is very small and results should be interpreted with caution. Close to 90% of the participants have lived in Merrimack for longer than two years.

### Exposures

Drinking contaminated water is the primary pathway by which Merrimack community members were exposed to elevated levels of PFAS [31]. Elevated levels of PFAS, in particular PFOA and PFOS, have been found in wells serving the public water supply (Merrimack Village District or MVD) and private wells, especially those close to the SGPP facility. Most respondents (90%) sourced their water from the public water system (MVD), and the rest had private wells. About 13% had installed water filtration system prior to concerns of PFAS contamination. About 6% of the residents in the study also participated in the NHDHHS blood test program that was made available to those living within 1.5-mile radius of the facility. There were others in the study that lived within the testing radius but had not participated in the blood testing program at the time the survey was conducted. Only a few people of those who had gotten their blood tested had received their test results back, and majority of them were still waiting for their results, some for over 8 months.

### Health outcomes

Nearly 28% of the participants said they experienced new health concerns since living in the community, and close to 20% reported multiple health conditions. Of all reports of health conditions, the most commonly reported were autoimmune conditions (28%) and cardiac disorders (22%). The third-most common health concern was reproductive (12%) and developmental disorders (8%). A wide range of cancers were also reported by close to 50 people. Altered liver and kidney functioning was a concern for close to 5% of the population. Frequencies of reports of health conditions (by category) are shown in Table 2.

A logistic regression analysis of *Health concerns since living in the community* compared to gender, age, occupational exposure, water source and years of residence in Merrimack showed that overall, women were twice as likely as men to report  health concerns since living in the community (OR = 2.09, CI 1.3–3.3). Reports of health concerns increased with the number of years lived in Merrimack. Those who have lived in Merrimack from 3 to 17 years (OR = 2.609, CI 1.0–6.9) and 18–30 years (OR = 2.588, CI 0.9–7.2) and over 30 years (OR = 4.274, CI 1.3–13.9) expressed health concerns two to four times compared to those who have lived in Merrimack for less than two years. A greater proportion of the respondents who worked at ChemFab or SGPP reported having health concerns compared to those who did not work at the facility (OR = 2.353, CI 0.4–12.2), but this association was not very strong. Table 3 provides a summary of the regression results.

### Multiple health concerns

Multiple health conditions include respondents who reported suffering from more than one health concern, such as both cardiac problems and reproductive disorders. As would be expected, the older population was twice as likely to report multiple health conditions. Multiple health problems were six times more likely to be reported by those between 19 and 44 years of age (OR = 6.495, CI 1.2–34.2), 17 times more likely to be reported by those between 45 and 64 years of age (OR = 17.656, CI 1.7–188.4), and 46 times more likely to be reported by those over 65 years of age (OR = 46.559, CI 1.9–1110.5) than those under eighteen years of age. Since the confidence intervals are very wide for these observations, this result needs to be further validated with studies with a larger sample size. Women were 72% more likely to report multiple health conditions (OR = 1.724, CI 1.0–3.1). Those who have worked at SGPP or ChemFab were also ten times as likely to report multiple health concerns (OR = 10.002, CI 0.7–138.2); however, this observation is based on a very small sample size and requires additional investigation to validate.

### Autoimmune disorders

Autoimmune disorders included thyroid problems, celiac disease, lower immune functioning, Crohn’s disease, Type 1 and II diabetes, lupus, rheumatoid arthritis, and psoriasis. Women were twice as likely to report autoimmune disorders (OR = 2.684, CI 1.6–4.6). Those who had water filtration were also close to twice as likely to report autoimmune disorders (OR = 1.977, CI 0.9–4.5).

### Cardiac disorders

The most commonly reported cardiac problems were high cholesterol and high blood pressure. Other cardiac problems reported included stroke, cardiomyopathy, left atrial myxoma, high triglycerides, high triglycerides, and atrial fibrillation, ventricular tachycardia, and heart palpitations. Reports of cardiac problems also increased with the number of years spent in Merrimack. Those who have lived in Merrimack for over 30 years were six times more likely to report cardiovascular disorders (OR = 6.163, CI 1.2–30.4) and those who have lived in Merrimack between 3-17 and 18-30 years were three times as likely to report cardiovascular problems compared to those who have been in Merrimack for less that two years. Those who have worked at SGPP (OR = 2.339, CI 0.2–26.3) or were on town water (OR = 2.028, CI 0.6–6.4) were also twice as likely to report cardiovascular problems, though these associations were weak.

### Respiratory problems

Respiratory problems reported include asthma, chronic obstructive pulmonary disease, and pulmonary embolism. There were no strong associations between respiratory problems and gender, age, occupational exposure, water source or years of residence in Merrimack. Interestingly, respiratory problems were reported at a higher rate among the younger population under 18 (10.9%), compared to those aged 19–44 years (2.4%) 46–64 (6.3%), and over 65 (3.5%). Those who have worked at ChemFab/SGPP were also four times more likely to report respiratory problems than those who had not worked at ChemFab/SGPP (OR 4.845, CI 0.3–70.1).

### Reproductive problems

Reproductive problems reported by female survey participants included preeclampsia, birthing problems, endometriosis, polycystic ovarian syndrome, menstrual cycle disruption, amenorrhea, dysmenorrhea, changes in reproductive development, changes in onset of puberty ovarian cancer, and breast cancer. Reports in males included, prostatitis, changes in reproductive development, enlarged prostate, testicular stones and cysts, testicular pain and inflammation and testicular cancer. Women were twice as likely to report reproductive problems (OR = 2.383 CI: 1.2–4.8) than men. Reproductive disorders also increased with age with those over 65 reporting reproductive disorders five times more likely than those under 18 years of age.

### Developmental disorders

Developmental disorders reported included: low birth weight, spina bifida, Asperger’s syndrome, sensory processing disorder, autism, ADHD, learning disabilities, Ehlers Danlos Syndrome, congenital cytomegalovirus, born with hydronephrosis, Atrial septal defect, food protein induced enterocolitis syndrome, Reynaud’s syndrome, tic disorder, and seizures. There was a negative correlation between age and developmental disorders. Those under 18 years of age were more likely to experience development disorders than those between 19 and 44 years of age (OR = 0.065 CI 0.0–0.9) and those over 65 (OR = 0.002 CI 0.0–0.8). Participants who had water filtration were also close to three times more likely to report developmental disorders (OR = 2.960 CI 0.7–12.8). The reports of developmental disorders also increased with the number of years lived in Merrimack. Residents who have lived in Merrimack for 18–30 (OR = 4.966 CI 0.6–42.9) and over 30 years (OR = 5.456 CI 0.3–90.6) were five times as likely to report developmental problems.

### Kidney problems

There were no strong associations with kidney problems and any other factor. Kidney problems (e.g. decreased kidney function, increased uric acid, and kidney stones) were twice as elevated for women (OR = 1.914 CI 0.7–5.2) and four times more elevated in 45–64 year olds (OR = 12.808 CI 1.4–120.1) and those over 65 years of age (OR = 4.717, CI: 1.04–2.83). Those who had no water filtration were also 74% more likely to report kidney problems compared to those with filtration (OR = 0.264 CI 0.1–2.1) but this observation was not strong.

### Liver problems

Liver problems, (e.g. increased liver enzymes, decreased liver functioning, liver lesions) increased with age among 19–44 year olds (OR = 2.189 CI 0.2–27.7) and three fold among 45–64 year olds (OR = 3.210, CI 0.1–117.2). Reports of liver problems were elevated among those who have worked at the SGPP facility (OR = 54.967, CI 4.3–692.6). Those who have been in Merrimack less than two years were also more likely to  report liver problems compared to those who have been living in Merrimack between 18 and 30 years (OR = 0.135 CI 0.0–0.9). Liver problems were also three times as likely to be reported among those who were on town water (OR = 3.135, CI 0.3–33.7).

### Cancer

Cancers reported included that of the breast, thyroid, prostate, lung, leukemia, kidney, esophagus, liver, brain, colon, cervix as well as lymphoma, melanoma and osteosarcoma. As it would be expected, cancer increased significantly with age, four times among 19–44 year olds (OR = 4.366, CI: 2.56–75.46), 62 times among 45–64 year olds (OR = 62.808 CI 1.8–2134.5) and 639 times among those over 65 years of age. The wider confidence intervals of these results, however, warrant further investigation. Those who had occupational exposures were four times more likely to report cancer (OR = 4.189 CI 0.2–99.2). No other demographic or exposure variables were associated with cancer.

## Discussion

This research provides a health profile of a population primarily located in Merrimack, New Hampshire, that has been exposed to PFAS contamination in drinking water. This survey represents the preliminary efforts by the community to compile and document health data in the Merrimack/Litchfield/Bedford area, and fills a crucial gap in documenting health outcomes connected to PFAS exposure that are not otherwise being tracked in this community. This survey is unique in representing the health information of children under 18 years of age as well as a small sample of people who have worked at the Chemfab/SGPP facility. This project was made possible because it was completely initiated by community members.

Since nearly all of the survey respondents are from Merrimack and adjacent communities close to the facility (See Fig. 1), the survey represents a population that has been exposed to elevated levels of PFOA through MVD drinking water. MVD water was found to be contaminated with PFAS in March 2016; close to 90% of the population were on town water and 9% on private wells. The latest testing results of wells (February 2019) showed PFOA at levels ranging from 8.4 to 22 ppt, and 1.3–2.5 ppt PFOS [74]. Since most wells (71%) tested in the Merrimack area have levels of PFAS above 10 ppt, and nearly a quarter of wells tested (23%) as of January 2017 exceeded the state’s 70 ppt drinking water cutoff [75], there is reason to believe that participants in this study with private wells may also have been exposed to elevated amounts of PFAS through drinking contaminated well water. Hence, in this survey, all the participants represent an exposed population and the range in exposure is primarily represented by the years living in the community and the age of the person.

Our results show that those who have lived in Merrimack longer and women have significantly more health concerns since living in Merrimack than those who have been in Merrimack for less than two years. Women also reported more associations with autoimmune disorders and reproductive problems, though the latter result may be nuanced by the fact that the conditions included in the reproductive category differ between men and women, and thus the rates of these gender-specific reproductive issues (e.g., preeclampsia, prostatitis) cannot be compared. The older population reported more correlations with multiple health conditions, reproductive disorders, kidney, liver problems and cancer even after controlling for age.

The younger population below 18 years of age also reported higher reproductive and developmental issues. The fact that 24% of youth under 18 years of age reported some type of reproductive health disorders (*n* = 11), over 56% (*n* = 26) of the those under 18 years of age had developmental problems, and 39% (*n* = 18) of those under 18 years of age reported autoimmune problems is a noteworthy finding that warrants further investigation. However, the small sample size of the population, and the clustering of self-reported observations of reproductive and developmental health issues into a single category, limits our ability to draw conclusions of any strong associations. These results though support the studies that show that PFAS have been implicated in a number of developmental, immunological, and reproductive health effects from early age through puberty. For instance, PFOA in mothers’ blood have been linked to slightly lower birth weight and/or head circumference in their babies [62, 76, 77] and birth defects [62]; higher ratios of PFOA in the blood of infants compared to their mothers [63, 78], children exposed to PFOA have shown to have weakened vaccine response [61] highlighting the vulnerability of infants and children to PFAS present in the environment. Observations of kidney disorders (16%, *n* = 4) in the youngest group, warrants attention as well. Some studies indicate that PFAS in children and adolescents may be associated with biomarkers of kidney function [79, 80]. Impacts of PFAS exposure to younger groups is of particular concern as infants, young children, and youth face unique dangers from PFAS exposure. Any additional health studies related to PFAS exposure in the Merrimack area should include a specific focus on infants, children, and youth.

This survey also represents a very small group of individuals who have worked at ChemFab/SGPP in the past and may have been ocupationally exposed to PFAS. This population may have had higher industrial exposure than non-occupationally exposed residents living in the community. However, the small sample size of this group makes it difficult to confirm any associations and further studies are needed to validate these results, which included a strong correlation with self-reported liver disease and higher observations of health concerns since living in the community, multiple health conditions, cardiovascular disorders, respiratory problems, and reproductive problems. Biomarkers of liver damage have been associated with PFOA exposure in the general population [77] and groups with higher exposure in the Ohio River Valley (C8 Studies) [81]; however, changes in liver biomarkers and liver disease are not consistently associated with PFOA exposure in occupational studies (reviewed in [38]). A second round of surveying that includes a greater number of occupationally exposed participants in the Merrimack area would be useful to clarify if liver problems are indeed a significant trend in this group.

A noteworthy pattern in these results is the overall parallel between the reported health conditions in this survey and conditions known to be associated with PFAS (and particularly PFOA) exposure. Among the study population as a whole, the three most common conditions reported fell into the categories of autoimmune, cardiovascular, reproductive, and developmental disorders. Immunotoxicity is an emerging area of concern regarding PFOA and PFOS exposure, as highlighted in a recent report by the National Toxicology Program [82] that determined that PFOA and PFOS were both “presumed to be” immune hazards to humans, primarily through evidence of suppressed antibody response. The report also noted “additional, though weaker evidence” that PFOA exposure is associated with increased autoimmune disease incidence in humans, and that PFOS suppresses disease resistance and immune function in animals. Elevated cholesterol has been consistently connected to PFAS exposure [39, 56, 58], and is a risk factor for multiple heart conditions. However, heart disease itself (e.g. coronary artery disease, myocardial infarction, angina, etc.) was found to have no probable link to PFOA exposure in the C8 study [83], and other studies report conflicting results (e.g. [84, 85]).

The results in this study show that among the participants of this survey, there are some significant occurrences of health concerns known to be connected to PFAS exposure that warrant further attention. However, several limitations are inherent in this survey design. These results only represent those exposed to PFAS contamination with no comparison to the control population, hence it is not possible to conclude from this analysis if the people in this community experience disproportionally higher health concerns compared to a less-exposed, control population. The study is also not based on a random sample of survey but has relied on volunteers and word-of-mouth to attract participation. It is also possible that those who were concerned about the PFAS contamination and health outcomes participated in this survey at a higher rate than those who were not concerned about PFAS-related health outcomes. However, the inclusion of an almost equal number of healthy participants (i.e., participants reporting no health conditions) reduces the impact that this type of bias may have on the results. Furthermore, this analysis was not controlled for any confounding factors such as smoking, family disease history, income, ethnicity, or any other factors. Finally, while it is reasonable to conclude that most or all of the survey participants were exposed to elevated levels of PFAS and especially PFOA, much remains unknown about their exact exposure levels over time, or about the variation in exposure levels within the study population.

For these various reasons, the results of this survey cannot be connected causally to PFAS exposure, nor are they generalizable beyond the study group. However, even with its limitations, this study provides a robust health profile of the participants, all of whom were likely exposed to elevated levels of PFAS through drinking water. There are a few observations that warrant further investigation and more immediate attention, especially: 1) elevated incidence of reproductive, developmental, autoimmune and kidney disorders among those under 18 years of age; 2) elevated levels of health concerns, multiple health concerns, autoimmune disorders, and reproductive disorders among women, 3) elevated levels of health concerns, multiple health conditions, cardiovascular, respiratory, reproductive, and liver disorders in those with industrial occupational exposures, and; 4) elevated incidence of health concerns, cardiovascular and developmental disorders among those who have been living in Merrimack for a long time versus newer residents.

This health survey was designed to collect data on a very broad range of health concerns in Merrimack, NH, in order to fill a crucial information gap as perceived by the community members on the health consequences of drinking PFAS-contaminated water. Results of this study are being used to advocate for access to medical monitoring, physician support, and additional health studies to follow up on specific concerns identified by this research. Actions taken by other states dealing with similar contamination issues continue to suggest a disparity between New Hampshire and other states in tracking health outcomes connected to PFAS exposure. For instance, it is notable that the New York Department of Health has developed an online health survey to gather health information for residents exposed to PFAS in drinking water in the exposed communities of Hoosick Falls, Petersburgh, and Newburgh, it has created a database of 1700 community health surveys [86]. Vermont moved quickly to work with the Centers for Disease Control to offer free blood testing to residents of Bennington, VT whose private wells were tested by the state (regardless of the test results) soon after PFAS contamination was discovered there [87]; Bennington College also enlisted former EPA officials to help design a community health survey to track health outcomes in the exposed community [88]. The Vermont legislature also continues to press for medical monitoring rights for citizens exposed to toxic substances, a right that courts in sixteen other states have affirmed [89].

Meanwhile, Merrimack residents continue to grapple with getting access to medical monitoring, physician support, and rigorous health studies as well as site remediation and clean up. A recent report noting that New Hampshire has the highest pediatric cancer rate in the country [90] has added a sense of urgency to investigate environmental burden of disease in these communities. Residents continue to engage with regulators and public utilities to address their concerns, for instance by voting overwhelmingly in favor of a recent proposal to raise over 15 million dollars for filtration of additional public wells, a cost that will be paid through a 79% increase in water utility rates [91]. Statewide regulation of PFAS in New Hampshire has moved forward, but still falls short of addressing next-generation PFAS. In January 2019, NHDES proposed MCL standards for four PFAS (PFOA, PFOS, PFNA, PFHxS), which could become the state’s first binding drinking water regulations to go into effect for PFAS chemicals [92]. Recent testing of raw materials and stack deposits at SGPP as well as dust from nearby residences also identified dozens of PFAS, including several novel compounds, still present in SGPP materials and emissions [18, 19, 21].

State and federal agencies should take note of this community survey—and the network of committed volunteers and collaborators that made it possible. They have laid the groundwork for the demands that local residents in the Merrimack area have been calling for—thorough tracking (biomonitoring, health guidance and physician support) and response to health concerns related to long-term exposure to PFAS-contaminated drinking water.

## Data Availability

The datasets used and/or analyzed during the current study are available from the corresponding author on reasonable request.

## Abbreviations

APFO: Ammonium pentadecafluorooctanoate
ATSDR: Agency for Toxic Substances and Disease Registry
MCFCW: Merrimack Citizens for Clean Water
MCL: Maximum Contaminant Level
MVD: Merrimack Village District
NHDES: New Hampshire Department of Environmental Services
NHDHHS: New Hampshire Department of Health and Human Services
PFAS: Per- and polyfluoroalkyl substances
PFHxS: Perfluorohexane sulfonic acid
PFNA: Perfluorononanoic acrid
PFOA: Perfluorooctanoic acid
PFOS: Perfluorooctane sulfonate
PTFE: Polytetrafluroethylene
